# Transient abnormal myelopoiesis with extramedullary involvement in a down syndrome preemie leading to an unresponsive course despite chemotherapy

**DOI:** 10.1016/j.lrr.2023.100381

**Published:** 2023-07-20

**Authors:** Saroja Devi Geetha, Ram Singh, Meira Shaham, Ninette Cohen, Kristin Sticco

**Affiliations:** a Department of Pathology and Laboratory Medicine, North Shore University Hospital and Long Island Jewish Medical Center, Donald and Barbara Zucker School of Medicine at Hofstra/Northwell Health, 2200 Northern Blvd, Suite 104, Greenvale, NY 11548, United States; b Cytogenetics Laboratory, Long Island Jewish Medical Center, Donald and Barbara Zucker School of Medicine at Hofstra/Northwell Health, United States

**Keywords:** Down syndrome, Transient abnormal myelopoiesis, Transient leukemia, Transient myeloproliferative disorder, Leukemia in Down syndrome, Megakaryoblastic leukemia, GATA1 mutation

## Abstract

**Introduction:**

Transient abnormal myelopoiesis (TAM) is a transient, clonal myeloproliferative disorder unique to Down Syndrome (DS) babies. It is characterized by increased peripheral blasts and presence of *GATA1* mutation. The clinical spectrum ranges from jaundice and hepatosplenomegaly to multi-organ failure and death. Here we present a case of a premature baby with DS diagnosed to have TAM with extramedullary involvement at birth who had a fatal outcome.

**Case report:**

A 30.3-week-old female fetus with DS had leukocytosis (WBC: 187.82 K/uL) with neutrophilia (ANC 27.65 K/uL), macrocytic anemia (RBC: 2.41 m/uL, Hb 8.8 g/dL, MCV 108.3, MCH 36.5, MCHC 33.7) and thrombocytosis (platelet count 361 K/uL) at birth. Liver panels demonstrated normal bilirubin levels with elevated liver enzymes (AST = 239 U/L, ALT = 216 U/L).

**Results:**

Peripheral smear showed marked leukocytosis with increased blasts (70%), nucleated RBCs, giant platelets, and megakaryocytic elements. Flow cytometry demonstrated two populations of cells: 20% myeloblasts and 26% dim CD45 CD34- cells. *GATA1* mutation was present. Based on these findings a diagnosis of TAM with extramedullary hematopoiesis was made. She received two cycles of cytarabine chemotherapy. Though her WBC levels reached a low of 18.93 K/uL, she developed multi-organ failure, eventually leading to death on day 45.

**Discussion:**

TAM is a transient condition resulting in disease resolution in around 80% of cases. Death is reported in 10% of cases. Risk factors associated with early death include prematurity, hyperleukocytosis, elevated bilirubin levels. Management of high-risk babies with chemotherapy is recommended to improve survival.

## 1 Introduction

Transient abnormal myelopoiesis (TAM) is a transient and clonal, myeloproliferative disorder recognized in babies with Down syndrome (DS). It is characterized by elevated white blood cell count (WBC) with increased peripheral blasts and the presence of a *GATA1* mutation. The clinical spectrum ranges from jaundice and hepatosplenomegaly to multi-organ failure and death. Most cases of TAM resolve spontaneously by three months of age. However, a few cases may progress to develop acute myeloid leukemia (AML); hence, TAM is considered a pre-leukemic condition. Here we present a case of a premature baby girl with DS diagnosed to have TAM with extramedullary involvement at birth who had a fatal outcome.

## 2 Case report

Owing to hydramnios and a low biophysical profile, a 30 and 3/7-week-old female fetus was delivered via urgent Cesarean-section. The mother is a 43-year-old with history of type II diabetes mellitus. Non-invasive prenatal screening was positive for Trisomy 21. Fetal ultrasonogram showed absent nasal bone, right hand clinodactyly, and hepatomegaly.

At birth, the baby had pale blue skin, poor muscle tone and respiratory effort with an APGAR of 1 which subsequently improved to 8, after 10 mins. Physical examination revealed low set ears, upward slanting eyes, flattened nasal bridge, thick nuchal skin, 2/6 soft ejection systolic murmur, enlarged liver palpable up to 4 cm below rib margin and enlarged spleen palpable up to 2.5 cm below rib margin.

**Fig. 1:**
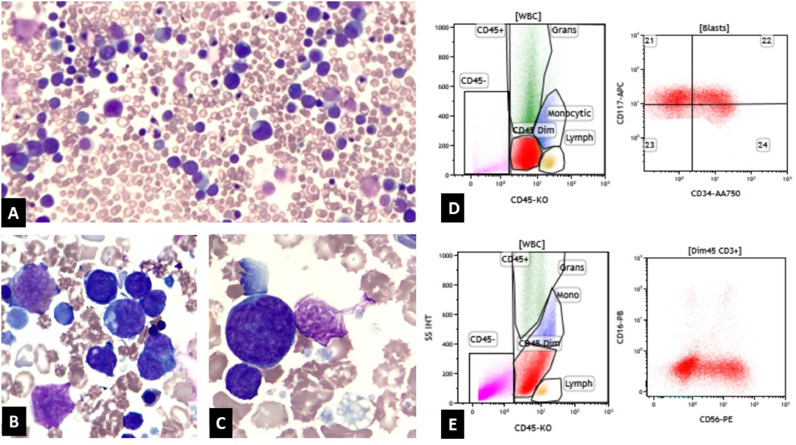
A: Peripheral smear showing leukocytosis with increased blast cells B & C: Peripheral smear showing blast cells and giant platelets. D: Flow cytometry demonstrating the myeloblast population, which are CD34 and CD117 positive. E: Flow cytometry demonstrating the second population of cells which are dim CD45, CD34- and partial CD56.

Complete blood count (CBC) revealed leukocytosis (WBC: 187.82 K/uL) with neutrophilia (ANC 27.65 K/uL), macrocytic anemia (RBC: 2.41 m/uL, Hb 8.8 g/dL, MCV 108.3, MCH 36.5, MCHC 33.7) and thrombocytosis (platelet count 361 K/uL) (Table 1). Peripheral smear examination showed marked leukocytosis with increased blasts (70%), nucleated RBCs, giant platelets, and megakaryocytic elements, both mature and immature (Fig. 1A, 1B, 1C). The liver panel demonstrated normal bilirubin levels with elevated liver enzymes (AST = 239 U/L, ALT = 216 U/L).

**Table 1:** Lab values depicting complete blood count, liver enzymes and tumor lysis markers.

	Birth	Post Exchange Transfusion	End of Cycle 1 Chemo	Few days following Chemo	Pre-Chemo 2	Post-Chemo 2
WBC* (K/ul)	187.82	23.93	26.39	57.89	73.37	18.93
RBC* (M/uL)	2.41	4.16	3.95	4.44	4.72	4.47
Hemoglobin (g/dl)	8.8	12.8	11.6	12.9	13.9	12.6
Hematocrit (%)	26.1	37.1	34.5	37.4	39.6	36.5
MCV* (fL)	108.3	89.2	87.3	84.2	83.9	81.7
MCH* (pg)	36.5	30.8	29.4	29.1	29.4	28.2
MCHC* (gm/dL)	33.7	34.5	33.6	34.5	35.1	34.5
Platelet Count (k/uL)	361	101	49	46	60	66
IANC* (k/uL)	27.65	9.66	10.70	43.04	65.16	16.76
AST* (U/L)	239	32	29	56	122	371
ALT* (U/L)	216	29	13	28	98	409
Potassium (mmol/L)	>10	2.5	4.3	4.4	4.8	6.2
Phosphate (mg/dL)	10.3	3.7	3.9	8.4	7.1	7
BUN* (mg/dL)	7	59	55	72	128	86
Creatinine (mg/dL)	0.58	2.15	1.13	0.77	1.18	0.66
Calcium (mg/dL)	9.1	8.8	11.2	10.2	10.2	9.9

* WBC: White blood cell count, RBC: Red blood cell count, MCV: Mean corpuscular volume, MCH: Mean corpuscular hemoglobin, MCHC: Mean corpuscular hemoglobin concentration, IANC: Instrument absolute neutrophil count, AST: Aspartate aminotransferase, ALT: Alanine aminotransferase, BUN: Blood urea nitrogen.

**Fig. 2:**
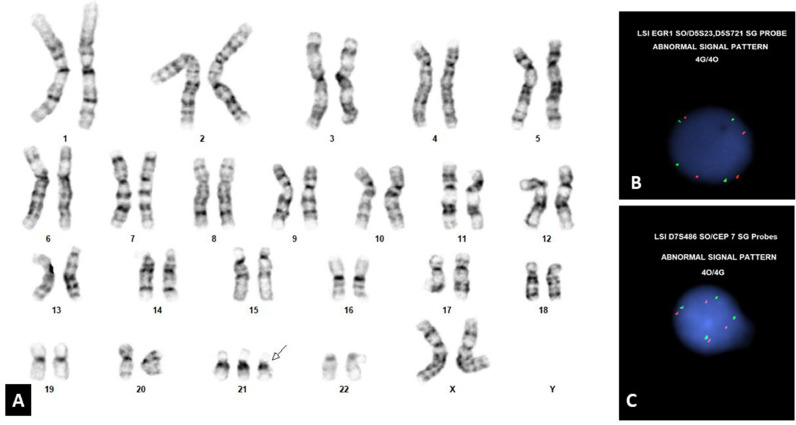
A: Karyotyping demonstrating additional copy of chromosome 21. B and C: FISH study demonstrating tetrasomy of chromosome 5 and tetrasomy of chromosome 7.

Chromosome analysis demonstrated an abnormal female karyotype with trisomy 21 (Fig. 2A), thereby confirming DS. *GATA1* mutation screening was positive. In this setting, the physical and laboratory findings confirmed TAM with extramedullary involvement of liver and spleen.

Further review of peripheral blood flow cytometry showed two cell populations. The first population was myeloblasts (20% of cells) (Fig. 1D), positive for partial HLA-DR.., CD38, CD34, CD117, partial CD33, CD7; negative for TdT, CD3 (surface and cytoplasmic), CD4, CD11b, CD13, CD15, CD19, CD64, CD123. The second population was a population of dim CD45 CD34- cells (26% of cells) (Fig. 1E), positive for CD38, CD117, CD7, partial CD56, CD36, CD41, CD42b, CD61; negative for TdT, HLA-DR.., CD3 (surface and cytoplasmic), CD4, CD11b, CD13, CD15, CD19, CD64, CD123. These findings are morphologically and immunophenotypically consistent with TAM.

Fluorescence *in situ* hybridization (FISH) studies on peripheral blood showed abnormal results for the AML and ALL panels. Specifically, four copies of chromosomes 5 and 7 and four copies of 4,10, and 17 were detected in 4.5% and 5.5% of cells, respectively (Fig. 2B, 2C). The signal pattern obtained for the *ETV6/RUNX1* probes, although negative for the translocation, was positive for gains of the *RUNX1* gene. Three signals of *RUNX1* were detected in 92.5% of cells, which is consistent with the constitutional karyotype of 47, XX, +21. In addition, four signals of *ETV6* (12p13) and six signals of *RUNX1* were detected in 5% of cells. Eight signals of *ETV6* (12p31) and three signals of *RUNX1* were detected in 2.0% of cells.

The presence of hyperleukocytosis (WBC: 187.82 K/uL) upscaled her TAM to high risk (WBC > 100 K/uL) requiring the need to initiate chemotherapy. Her clinical course was further complicated by disseminated intravascular coagulation (DIC) and hyperkalemia, and hyperuricemia due to tumor lysis syndrome. In order to temporarily reduce the leukocyte burden, she underwent double volume exchange transfusion and received medical management for tumor lysis syndrome and DIC.

With these measures WBC and tumor lysis markers began to downtrend (Table 1), but she remained coagulopathic with continuing evidence of extramedullary hematopoiesis. Hence, low dose cytarabine therapy was started, twice daily for 7 days. Her WBC following cycle 1 was 26.39 K/uL.

Three days following cycle one completion, her WBC began to rise further reaching 50Ks, (Table 1) for which she received another exchange transfusion. During the same period, her respiratory status deteriorated requiring intubation and ventilatory support. She also had a prolonged period of severe abdominal distension, ascites and hypotension concerning for sepsis, soon developing into renal failure. Her WBC continued to rise further and remained symptomatically coagulopathic with worsening of liver enzymes. Outweighing the risks and benefits, a decision was made to initiate the 2nd cycle of chemotherapy for 7 days, following which her WBC reached 18.93 K/uL which was the lowest WBC level for her (Table 1).

Despite extra-ordinary management efforts, her lungs, kidneys, and liver continued to deteriorate, resulting in multi-organ failure, eventually leading to death on Day 45.

## 3 Discussion

Children with DS are at risk for developing acute leukemias. A pre leukemic condition, known as TAM, is exclusively seen in DS babies. It is also known as transient leukemia or transient myeloproliferative disorder and was first reported in 1954. [1] It presents within the first few days of life and is characterized by a transient increase in the circulating blast cells. Pathogenesis behind development of TAM is believed to arise in-utero in the hematopoietic cells of the liver. [2,3] Two key events are required for TAM to occur: Trisomy 21 and mutation of *GATA1* gene. *GATA1* is a hematopoietic transcription factor, involved in the development and maturation of erythrocytes and megakaryocytes and is located on chromosome X. [4,5] Clinical manifestations vary, with jaundice, hepatomegaly, splenomegaly, pleural effusion, and skin rash being the most common presentations [6]. Laboratory findings include leukocytosis due to increase in neutrophils, metamyelocytes, basophils and monocytes with increased blast cells on the peripheral smear. These blast cells are usually megakaryoblast appearing. Silent TAM has been reported wherein cases lack clinical and hematological symptoms but have *GATA1* mutations [7]. Most cases of TAM resolve spontaneously by 3 months of age [8]. In few, the blasts can infiltrate extramedullary sites and progress to develop organ dysfunction such as liver fibrosis, heart failure and renal failure, eventually leading to multi-organ failure and death [9]. Though factors leading to such worse outcomes have not been fully studied, it is found that early gestational age, hyperleukocytosis and elevated bilirubin and liver enzymes could lead to poor outcomes [3,9].

The blast cells of TAM are positive for CD33, CD13, CD38, CD117, CD34, CD7, CD56, CD36, CD71, dim CD4 [10,11].

Currently, the presence of massive hepatomegaly defined as extending below the umbilicus along with end-organ failure and or extremely high WBC counts warrants treatment [8,3].. Low dose cytarabine is the drug of choice and has been found to reduce the number of peripheral blasts and provide symptomatic relief. At present, there are no well-developed management criteria or guidelines. Our case received two cycles of low dose cytarabine, however, the outcome was still unfavorable, probably due to the presence of hyperleukocytosis and liver infiltration along with cardiopulmonary and renal failure.

TAM is a pre-leukemic condition with 20% of cases developing AML, usually of the acute megakaryoblast type, associated with DS (ML-DS) in their early childhood. It is usually preceded by a period of resolution characterized by absence of clinical symptoms and normalization of blood and laboratory parameters. Some cases of TAM can directly progress to ML-DS without resolution. Thrombocytopenia and pleural effusions are associated with increased progression to ML-DS [10].

Recently, few cases of TAM have been diagnosed during the prenatal period, especially during the second and third trimester. Hepatomegaly is the most common ultrasound finding in these cases. Other findings include hydrops fetalis, pericardial effusion and variations in amniotic fluid index. Our case had hepatomegaly and hydramnios on fetal ultrasound hinting that she had TAM in-utero. Supportive therapy in the form of intrauterine fetal blood transfusions have been tried to manage anemia and thrombocytopenia in such cases but the outcomes have been mixed. [12]

## 4 Conclusion

TAM is a pre-leukemic condition in babies with DS. Most cases of TAM resolve spontaneously by 3 months of age. However, a few cases may progress to develop AML. Chemotherapy with low dose cytarabine is currently advocated for TAM with life threatening symptoms. In our case, management with double volume exchange transfusion, medical management for tumor lysis syndrome and DIC, and low dose cytarabine therapy was used to manage the case. Though there were periods of laboratory improvement in our case, the baby continued to deteriorate clinically and had a fatal outcome. The case highlights the aggressive nature of TAM and its potential for fatal outcomes.

### 4.1 Informed consent

Based on the institutional IRB policies, consent is not required for case reports. Furthermore, all patient details have been de-identified and no images pertaining to identity of this case is used in this paper.

## Declaration of Competing Interest

None.
